# Intraspinal microstimulation of the ventral horn has therapeutically relevant cross-modal effects on nociception

**DOI:** 10.1093/braincomms/fcae280

**Published:** 2024-08-19

**Authors:** Maria F Bandres, Jefferson L Gomes, Jacob Graves McPherson

**Affiliations:** Department of Biomedical Engineering, Washington University in St. Louis, St. Louis, MO 63130, USA; Program in Physical Therapy, Washington University School of Medicine, St. Louis, MO 63108, USA; Program in Physical Therapy, Washington University School of Medicine, St. Louis, MO 63108, USA; Department of Biomedical Engineering, Washington University in St. Louis, St. Louis, MO 63130, USA; Program in Physical Therapy, Washington University School of Medicine, St. Louis, MO 63108, USA; Department of Anesthesiology, Washington University School of Medicine, St. Louis, MO 63108, USA; Department of Anesthesiology, Washington University Pain Center, Washington University School of Medicine, St. Louis, MO 63108, USA; Program in Neurosciences, Washington University School of Medicine, St. Louis, MO 63108, USA

**Keywords:** spinal cord injury, movement impairments, neuropathic pain, neuromodulation, neurorehabilitation

## Abstract

Electrical stimulation of spinal networks below a spinal cord injury is a promising approach to restore functions compromised by inadequate and/or inappropriate neural drive. The most translationally successful examples are paradigms intended to increase neural transmission in weakened yet spared descending motor pathways and spinal motoneurons rendered dormant after being severed from their inputs by lesion. Less well understood is whether spinal stimulation is also capable of reducing neural transmission in pathways made pathologically overactive by spinal cord injury. Debilitating spasms, spasticity and neuropathic pain are all common manifestations of hyperexcitable spinal responses to sensory feedback. Whereas spasms and spasticity can often be managed pharmacologically, spinal cord injury-related neuropathic pain is notoriously medically refractory. Interestingly, however, spinal stimulation is a clinically available option for ameliorating neuropathic pain arising from aetiologies other than spinal cord injury, and the limited evidence available to date suggests that it holds considerable promise for reducing spinal cord injury-related neuropathic pain, as well. Spinal stimulation for pain amelioration has traditionally been assumed to modulate sensorimotor networks overlapping with those engaged by spinal stimulation for rehabilitation of movement impairments. Thus, we hypothesize that spinal stimulation intended to increase the ability to move voluntarily may simultaneously reduce transmission in spinal pain pathways. To test this hypothesis, we coupled a rat model of incomplete thoracic spinal cord injury, which results in moderate to severe bilateral movement impairments and spinal cord injury-related neuropathic pain, with *in vivo* electrophysiological measures of neural transmission in networks of spinal neurons integral to the development and persistence of the neuropathic pain state. We find that when intraspinal microstimulation is delivered to the ventral horn with the intent of enhancing voluntary movement, transmission through nociceptive specific and wide dynamic range neurons is significantly depressed in response to pain-related sensory feedback. By comparison, spinal responsiveness to non-pain-related sensory feedback is largely preserved. These results suggest that spinal stimulation paradigms could be intentionally designed to afford multi-modal therapeutic benefits, directly addressing the diverse, intersectional rehabilitation goals of people living with spinal cord injury.

## Teaser

Bandres *et al*. report that spinal stimulation intended to facilitate voluntary movement following spinal cord injury simultaneously depresses overactive nociceptive transmission while preserving non-nociceptive transmission. These findings suggest that spinal stimulation-based therapies could be configured to provide multi-modal rehabilitation benefits, here applied to movement impairments and neuropathic pain.

## Graphical Abstract

**Graphical Abstract:**
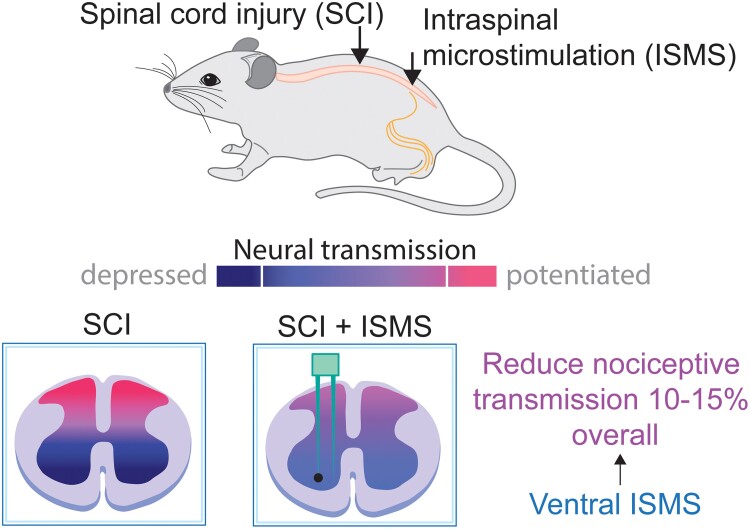

## Introduction

Empirical data and computational modelling support the view that electrical spinal stimulation non-specifically increases net spinal sensorimotor excitability via direct recruitment of large-diameter, low-threshold afferent fibres and trans-synaptic activation of low-threshold interneurons.^1-9^ This diffuse excitation is sculpted into functionally relevant movement commands by inhibitory proprioceptive networks intercalated amongst spinal motor pools prior to its integration by motoneurons.^2,5^ In this way, task specificity is not afforded by the stimulation itself but rather by the state of the reciprocally organized propriospinal networks during stimulation-enabled movements.^2,5^

However, recent studies have hinted that the neuromodulatory profile of electrical spinal stimulation may be more nuanced than this canonical understanding. In neurologically intact rats, direct electrical stimulation in the ventral horn (VH) has revealed a different set of actions: a predictable increase in net spinal motor excitability coupled with a net decrease in spinal responsiveness to nociceptive cutaneous feedback and minimal effects on non-nociceptive cutaneous transmission.^10^ Such a profile would be highly advantageous for spinal cord injury (SCI)-related applications, because reduced voluntary movement capacity after SCI is frequently accompanied by pathologically elevated spinal responses to sensory feedback resulting in SCI-related neuropathic pain (SCI-NP), spasms and spasticity.^11-14^ By differentially rebalancing sensorimotor excitability in networks below the lesion, such a neuromodulatory profile would hold considerable promise for delivering multi-modal therapeutic benefits.

The extent to which spinal stimulation intended to enhance voluntary movement can depress nociceptive transmission while preserving non-nociceptive transmission in the chronically injured spinal cord is un-established. Central to this question is the recognition that sensory-dominant regions of the spinal cord often become hyperexcitable after SCI, owing to a mechanistically enigmatic sequelae of maladaptive plastic changes during the chronification process.^15-17^ As such, spinal stimulation runs the risk of exacerbating already overactive responses to nociceptive sensory feedback while concomitantly increasing responsiveness to non-nociceptive sensory feedback and inadvertently leading to spasms and/or spasticity.

Here, we test the hypothesis that spinal stimulation intended to enhance transmission in movement-related pathways—specifically, intraspinal microstimulation (ISMS) delivered directly to the VH—depresses nociceptive neural transmission while preserving non-nociceptive transmission in rats with moderate to severe sensorimotor impairments secondary to chronic SCI. We find that movement-targeted ISMS retains the ability to reduce spinal responsiveness to nociceptive transmission through nociceptive specific (NS) and wide dynamic range (WDR) neurons below the lesion. These results were surprisingly modality specific, having little effect on non-nociceptive transmission. Furthermore, they could not be explained by overt ISMS-driven changes in overall segmental or regional excitability. These findings provide a translational roadmap for developing a new class of spinal stimulation-based therapies specifically intended to afford multi-modal therapeutic benefits—here, applied (but not limited) to simultaneous rehabilitation of movement impairments and SCI-NP.

## Materials and methods

### Study design

We studied the effects of ISMS intended to enhance transmission in movement-related pathways on spinal sensory transmission in the chronically injured spinal cord. All procedures were approved by the Institutional Animal Care and Usage Committee of Washington University in St. Louis. The study included 15 adult male Sprague–Dawley rats (∼400–550 g) with moderate to severe sensorimotor deficits following a midline spinal contusion injury at the T8/T9 vertebral border (Infinite Horizon Impactor, IH-04000; Precision Systems and Instrumentation, LLC; 200 kilodynes, 0-s dwell time). It also included one neurologically intact rat, which was used only as a qualitative comparison for visualization/contextualization.

### Animal care and behavioural assessments

After the SCI procedure, animals were transferred to recovery housing, which included a thermal pad to maintain core temperature (∼37°C). Heart rate, blood pressure, respiration rate and SpO2 were continuously monitored (Kent Scientific, Inc.) until arousal. Nutritional supplements, electrolyte replenishers (Bio-Serv) and water with antibiotic (enrofloxacin 0.5 mL/kg) and sweetener were provided to the animals during their recovery. Weight loss/gain, righting reflexes, forelimb and head/neck motor control, coat quality, demeanour, hydration and urine composition were closely monitored for at least the first 2 post-operative days. Each animal's bladder was manually expressed ≥ 2×/day for 7 days after the injury or until voiding reflexes returned spontaneously.

We assessed all animals for behavioural signs of below-level neuropathic pain at least twice per week beginning 1-week post-SCI and continuing until the terminal electrophysiological experiment (at ≥6 weeks after SCI). Common signs of SCI-NP in this contusion model include the presence of mechanical allodynia and/or hyperalgesia in dermatomes below the lesion. Our outcome measures included behavioural responses to mechanical probing of the L5 dermatomes ipsilateral and contralateral to the electrode implant.

Nociceptive reflex responses were based on the mechanical threshold (measured in grams of force) for eliciting an aversive withdrawal reflex on the plantar surface of each hindpaw using the up-down method.^18,19^ Forces were applied using small rigid probes, similar in profile to a micropipette tip, coupled to a von Frey system instrumented with a precision load cell (Electronic von Frey, IITC Inc.). Ten measurements were completed in each hindpaw per session, and the highest and lowest scores were discarded; the remaining thresholds were averaged. One animal was excluded from von Frey testing because it could not voluntarily move the hindpaw from the probe due to severe motor impairment.

Additional behavioural responses to noxious and innocuous cutaneous sensation included vocalizations during mechanical probing of the L5 dermatome as well as attendance to the involved hindpaw as evidenced by turning the head and neck towards it and/or attempting to reach it with the forelimb(s). To reduce the well-known confounds of stoicism in prey animals, increased stress associated with temporary transfer to the behavioural testing arena and non-pain-related hyperreflexia—all of which can alter attendance to routine handling, movements and/or noise—rats were allowed at least 20 min to acclimate to the testing environment prior to assessment.

The presence of an SCI-NP state was established by a ≥50% reduction in withdrawal threshold compared with an animal's pre-SCI baseline and/or clear cognitive signs of distress during innocuous exploration of the dermatome.

### Surgical procedure for electrophysiology experiments

In a terminal electrophysiological experiment at least 6 weeks after SCI, we recorded extracellular neural activity throughout the dorsoventral extent of the L5 spinal grey matter while inducing natural nociceptive or non-nociceptive feedback before, during and after 30-min epochs of sub-motor threshold ISMS delivered to the L5 spinal VH. Sensory feedback was induced by application of graded pressure to the most sensitive receptive field of the glabrous skin of the hindpaw ipsilateral to the implanted microelectrode array (MEA).

Anaesthesia was induced with inhaled isoflurane (∼1–3% O_2_, flow rate: 1–2 L/min), which was discontinued upon uptake of urethane (1.2 g/kg i.p.). Deep surgical plane anaesthesia was maintained with boosts of urethane as needed (0.2 g/kg i.p.). Urethane does not suppress vital signs (e.g. respiration and heart rate) to the same extent as isoflurane, and thus we supplemented these physiological monitors with continuous measurements of blood pressure as well as frequent assessment of withdrawal and blink reflexes. That being said, urethane preserves the excitability of spinal nociceptive and sensorimotor reflex pathways,^10,20^ which motivated its use here.

After the animals were deeply anaesthetized, the back and hindlimbs were shaved. Then, a ∼5-cm midline incision was made over the vertebral column on the shaved region. The exposed tissues and musculature were dissected, and a bilateral T13-L2 laminectomy was performed under magnification (Leica Microsystems, Inc.). For the duration of the procedure, the animal's temperature was maintained at ∼37°C using a thermal pad, and heart rate, blood pressure, respiration rate and SpO2 were continuously monitored (Kent Scientific, Inc.). Warmed lactated Ringer's solution (5 mL) was administered subcutaneously every 2 h to prevent dehydration.

After the laminectomy, animals were transferred to an anti-vibration air table (Kinetic Systems, Inc.) enclosed by a Faraday cage. Vertebrae rostral and caudal to the laminectomy were clamped with locking forceps connected to a custom, multi-degree-of-freedom fixation frame. The animals’ abdomen was raised using the fixation frame to attenuate chest cavity and spinal movements during respiration cycles. The spinal meninges were then incised rostrocaudally and reflected. The exposed spinal cord was continually bathed in homoeothermic ringer solution, and the MEA was implanted.

### Electrophysiology

**Figure 1:**
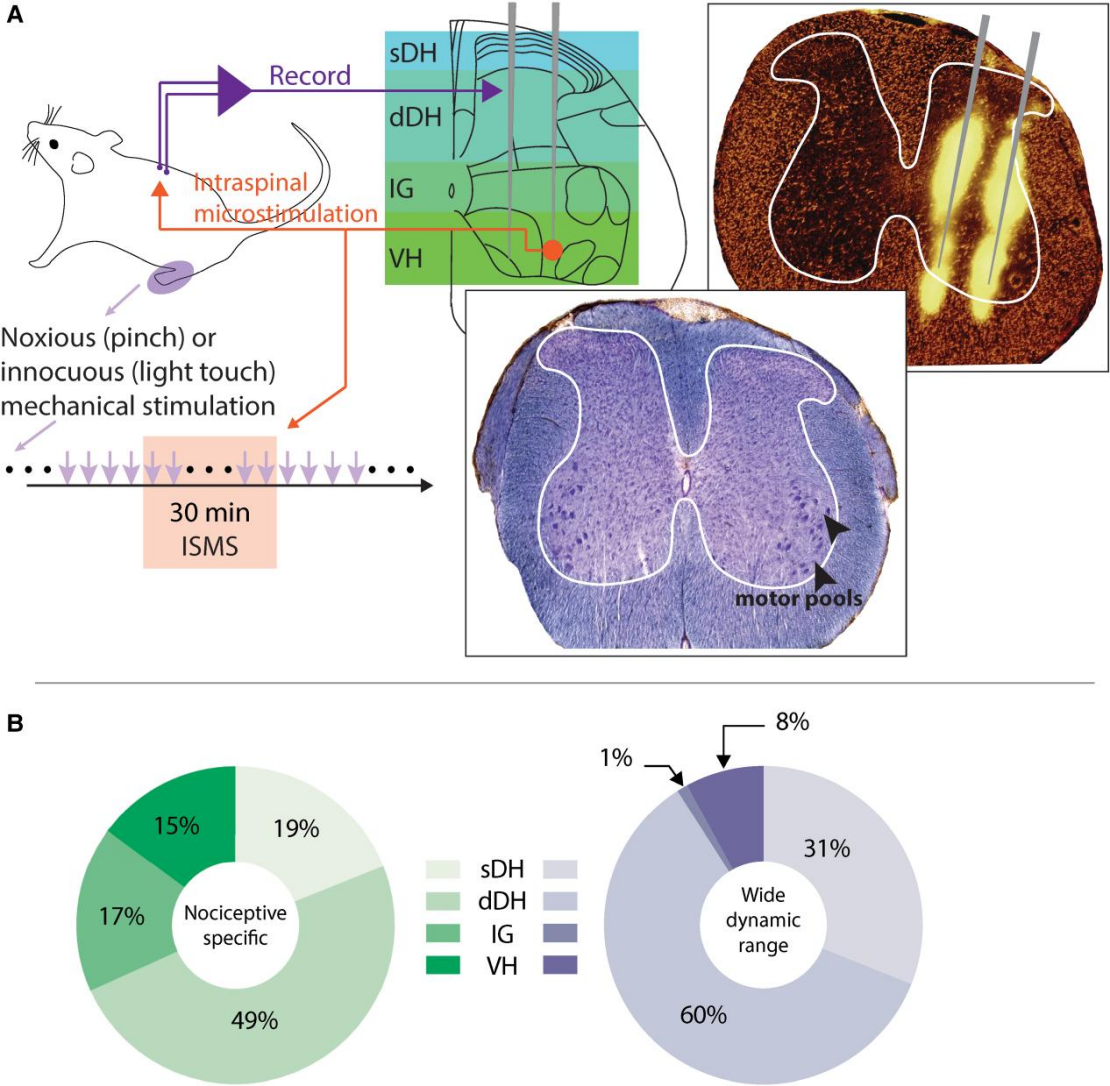
**Intraspinal MEAs enable *in vivo* characterization of sensorimotor neural transmission during VH spinal stimulation.** (**A**) Clockwise from top: 32-channel MEAs were implanted perpendicular to the midline at the L5 dorsal root entry zone. MEA coverage extended from the sensory-dominant dorsal horn to the motor-dominant VH; sDH, dDH, IG and VH. Top right: histological image of electrode tracks, with fluorescent-labelled MEA illuminated by RFP filtered light (EVOS RFP 2.0 Light Cube, wavelength: Ex: 542/20 Em: 593/40; Thermo Fisher Inc.,). Bottom center: histological image double-stained for cell bodies and myelin, highlighting the location of motor pools. Bottom left: mechanical stimulation was delivered to the plantar surface of the ipsilateral hindpaw before, during and after sub-motor threshold ISMS. (**B**) Anatomical distribution of functionally classified neurons (*N* = 15 rats). Left: NS neurons; Right: WDR neurons.

A single MEA was implanted into the spinal cord at the L5 dorsal root entry zone (Fig. 1A). Each array consisted of two parallel shanks, and both shanks contained 16 discrete, vertically aligned electrodes (area: 177 μm^2^; inter-electrode spacing: 100 μm; NeuroNexus Inc., A2 × 16). The tips of both shanks were sharpened to aid insertion. All MEAs were custom electrodeposited with activated platinum–iridium to lower impedance and increase charge capacity (impedance: 4–10 KΩ; Platinum Group Coatings, Inc.). Prior to implantation, MEAs were coated with 1,1′-dioctadecyl-3,3,3′,3′-tetramethylindocarbocyanine perchlorate (Sigma-Aldrich, Inc.) to aid post-mortem histological localization of the electrode tracks (as in Fig. 1A, top right panel).

For implantation, the MEA was oriented perpendicular and just lateral to the midline and positioned directly above the L5 dorsal root entry zone. Subsequently, it was lowered until the bottom-most electrode on each shank was lightly nested amidst the dorsal roots. We then probed the glabrous skin of the ipsilateral hindpaw while monitoring dorsal root potentials in real time with visual and audio feedback. If dermatome mapping evoked clearly correlated dorsal root potentials, MEA insertion began as detailed below. However, if no dorsal root potentials were evident during mapping or if the most responsive portion of the dermatome was not located on the plantar surface of the hindpaw, the MEA was repositioned, and mapping began anew.

Once the initial implant site was established, the MEA was slowly advanced into the spinal cord. Insertion was paused every ∼25–50 μm to minimize shear and planar stress on the neural tissue, which is particularly important for preserving the viability of the small, superficially located neurons in Laminae I–III. When the deepest electrodes of the MEA reached the most superficial border of the deep dorsal horn (dDH) (∼400–500 μm deep to the surface), we again mapped the L5 dermatome while monitoring neural transmission in real time. If clearly correlated multi-unit neural activity was evident during exploration of the desired receptive field, insertion continued. Conversely, if the most sensitive receptive field had shifted from the glabrous skin of the hindpaw to another region of the L5 dermatome, the MEA was withdrawn and repositioned. When fully inserted, the ventral-most electrodes were positioned ∼1600–1800+ μm deep to the surface of the spinal cord, corresponding to the motor pools of the VH, and the dorsal-most electrodes were ∼100–200 μm deep to the surface. Electrode placement was confirmed intra-operatively during motor threshold determination (below). Once insertion was complete, we again mapped the L5 dermatome. Electrode arrays were not moved after full implantation.

### Motor threshold determination and ISMS parameters

After MEA implantation, we determined the resting motor threshold for each animal. We delivered single pulses of charge balanced current (cathode leading, 200 μs/phase, 0-s inter-phase interval) to the electrodes located in the deepest regions of the VH. Current magnitude was increased in 1-μA steps until a twitch was detected in the L5 myotome (toe twitch on ipsilateral hindpaw). Then, we reduced the current intensity in 1-μA steps until the twitch was undetectable. The lowest current intensity at which a twitch was detected, across electrodes located in the VH, was taken as the resting motor threshold. The specific electrode on which the threshold was established was used to deliver ISMS on all subsequent trials.

Movement-targeted ISMS was delivered as a series of discrete, cathode-leading pulses at 7 Hz, 200 μs/phase, 0-s inter-phase interval and 90% of resting motor threshold (typically ∼3–12 μA/phase). These ISMS parameters are consistent both with values that induced functionally meaningful enhancements of spinal motor output below a chronic SCI^21^ and with stimulation intensities (e.g. 80–90% of resting motor threshold) commonly used in studies of epidural spinal stimulation parametrized to reduce neuropathic pain.^22,23^

### Data acquisition/trial structure

Trials had the following general sequence: (i) 1 min of innocuous mechanical stimulation of the receptive field, delivered as a series of light touches of the most sensitive region of the receptive field; (ii) 1 min of spontaneous activity (i.e. no mechanical stimulation of the receptive field); (iii) 3–5 min of noxious mechanical stimulation of the receptive field, delivered as a ∼1- to 2-s pinch of the most sensitive region of the receptive field every 30 s; (iv) 30 min of movement-targeted ISMS combined with phasic noxious mechanical stimulation of the receptive field; (v) 3–5 min of noxious mechanical stimulation of the receptive field (as above, in iii); (vi) 1 min of spontaneous activity; and (vii) 1 min of innocuous mechanical stimulation of the receptive field (as above, in i).

This trial structure allowed assessment of potential modulatory actions of ISMS on spinal nociceptive and non-nociceptive transmission. The 30-min ISMS duration was selected to mirror a previously published report of VH ISMS on spinal nociceptive transmission in neurologically intact rats.^10^ In that study, 30 min was found to be more efficacious at reducing nociceptive transmission than 2, 5 or 10 min of VH ISMS. It is also more comparable with the durations of stimulation that would be utilized for clinical pain management and household or community ambulation.

### Statistical analyses

Raw, multi-unit neural data were pre-processed offline to remove non-physiological features (e.g. electrical noise and artefacts caused by respiration cycles) (The MathWorks, Inc.). Cleaned multi-unit data were then decomposed into spike trains of individual neurons using the well-validated wavelet-based spike sorting algorithm, ‘wave_clus’.^10,20,24,25^ Discrimination parameters were as follows: band-pass filter: 1 Hz to 15 kHz; minimum detection threshold: 4 SDs from mean; maximum detection threshold: 25 SD; detection thresholds on both positive and negative deviations; filter order for detection: 4; filter order for sorting: 2; spike trains were then analysed manually to remove any errors associated with the decomposition (e.g. predominance of inter-spike intervals < 2 ms, non-physiological shape of the action potential or inappropriate action potential duration). Neurons not passing the manual verification stage were discarded (typically 5–20 total neurons for a given animal, leaving ∼70 well-isolated neurons per animal; see Results).

All statistical analyses were based upon the instantaneous discharge characteristics of individual NS, WDR and other, functionally non-classified (NC) neurons during nociceptive or non-nociceptive transmission before, during and after ISMS. Specifically, a wavelet-based spike sorting algorithm^24^ decomposed multi-unit extracellular neural activity into a series of spike times of discrete neurons. Neurons exhibiting increased discharge rates during nociceptive and non-nociceptive sensory transmission were classified as WDR, neurons whose discharge rate increased only during nociceptive transmission were classified as NS, and all other neurons were termed NC, regardless of whether they were tonically or phasically active.

For inclusion in quantitative analyses, NS and WDR neurons were required to manifest a mean pre-ISMS discharge rate of >6 Hz during periods of induced nociceptive transmission and a mean discharge rate of >0.11 Hz during and following ISMS; NC neurons were required to manifest a nominal mean discharge rate of >0.11 Hz throughout a given trial. These criteria ensured inclusion only of neurons that could be readily tracked across all segments of a trial. However, in so doing, they also excluded from consideration neurons that were fully de-recruited during or following ISMS. Thus, our numerical estimates of the proportion of neurons exhibiting depressed responses to nociceptive transmission are likely an underestimate of the actual impact of ISMS.

Discharge characteristics were characterized in two primary ways: animal level and neuron level. This dual approach is intended to capture the heterogeneity associated with spinal contusion injuries such as that used here. For animal-level analyses, discharge rates were averaged within each animal prior to averaging across a given group. Groups included the entire cohort of animals, the subgroup of animals without SCI-NP, and the subgroup with SCI-NP. For neuron-level analyses, the mean peak discharge rate for each neuron was considered to be independent; that is, animal was not included as a grouping variable *per se*. However, for neuron-level subgroup analyses, animal was implicitly used as a grouping variable insofar as it was necessary to pool neurons from animals with or without SCI-NP, respectively.

Animals were not randomized, although it was not possible to know *a priori*, which animals would develop SCI-NP following injury. Behavioural analyses were conducted by an experimentalist not involved in terminal electrophysiological experiments, and it was not possible to determine during a terminal electrophysiological experiment whether a given animal did or did not exhibit behavioural signs of SCI-NP.

Mean discharge rate was defined as the grand mean of the maximum instantaneous discharge rate of each neuron across all instances of mechanical probing of the dermatome for a given time point. For example, if a pre-ISMS baseline epoch of nociceptive transmission contained 10 pinches of the receptive field, the peak discharge rate during each pinch was averaged for each neuron of a given type, after which those mean neuron-level rates were averaged to arrive at an animal-level grand mean.

Comparisons of population discharge rates across time (relative to ISMS) utilized one-way repeated measures ANOVAs. Separate ANOVAs were computed for NS, WDR and, in the case of overall excitability, pooled NS, WDR and NC neurons. For all comparisons, the independent variable was time point relative to ISMS and the dependent variable was mean discharge rate per animal. *Post hoc* analyses compared mean discharge rates before ISMS (during nociceptive transmission) with each subsequent time point during and following ISMS, and Bonferroni correction was used to control family-wise error rate. WDR discharge rate during non-nociceptive transmission included only pre-ISMS and post-ISMS time points. Thus, paired, two-tailed *t*-tests were used to compare these rates. Descriptive statistics presented in the text and figures display mean + 95% confidence interval unless otherwise noted; specific *P*-values are indicated if <0.1; otherwise, comparisons are labelled ‘n.s.’. Statistical analyses were performed using GraphPad Prism software (v9).

A power analysis was performed *a priori* (G*Power 3.1) using data extracted from a recently published report of VH ISMS on nociceptive and non-nociceptive transmission in neurologically intact rats.^10^ Those data allowed determination of a presumptive ‘best-case scenario’ effect size for ISMS-associated depression of discharge rates during nociceptive transmission. Achieved effect size in that study for one-way repeated measures ANOVAs with the same independent and dependent variables as those used here was estimated to be 0.48 considered a medium effect by standard conventions. Using that effect size, *α* = 0.05, power (1 − *β*) =0.9, and a moderate correlation amongst repeated measures (0.6; range of 0–1, where the repeated measures are the mean discharge rate per rat across time points), we estimated that seven animals would be required.

However, recognizing both the heterogeneity associated with contusion models of SCI as well as the proclivity of the dorsal horn to become hyperexcitable post-SCI, we expected to realize a smaller effect size. Thus, we chose a more conservative estimate of effect size, 0.3, corresponding to a small standardized effect. Using this effect size instead (while carrying forward the other parameters), the required sample size was determined to be 15 animals.

## Results

Seven of the fifteen animals exhibited nocifensive withdrawal reflexes and cognitive responses to von Frey filament testing consistent with SCI-NP, seven of the animals did not, and one animal was unable to withdraw its hindlimbs from the filament and therefore was not assessed for SCI-NP (note that the neurologically intact rat was not included in statistical analyses). On average, we identified 10 (±2) NS neurons and 12 (±1) WDR neurons per animal. We also identified an additional 50 (±4) well-isolated yet functionally indeterminant neurons per animal (i.e. NC neurons). We broadly categorized their locations as falling into one of four regions of the L5 grey matter: superficial dorsal horn (sDH), dDH, intermediate grey (IG) and VH. The average anatomical distribution of NS neurons per animal was sDH: 2 (±1), dDH: 5 (±1), IG: 2 (±1) and VH: 1 (±1); for WDR neurons, the average anatomical distribution was sDH: 4 (±1), dDH: 7 (±1), IG: 0 and VH: 1 (±1); and for NC neurons, the distribution was sDH: 3 (±1), dDH: 19 (±2), IG: 15 (±1) and VH: 13 (±1) neurons (Fig. 1B).

### Movement-targeted ISMS depresses nociceptive transmission in the injured spinal cord

**Figure 2:**
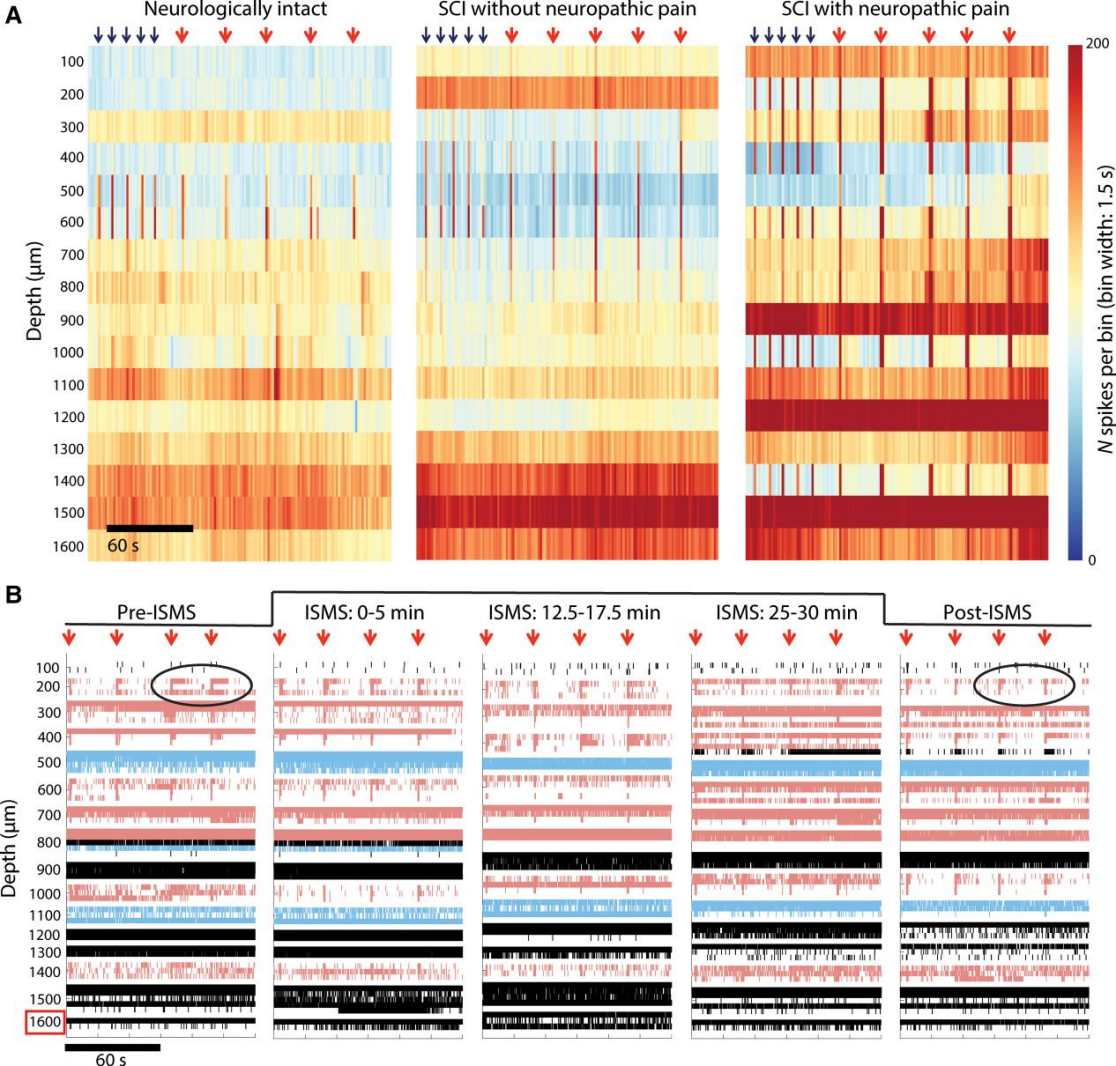
**VH ISMS leads to widespread modulatory actions that counter sensory hyperexcitability post-SCI.** (**A**) Time-resolved histograms of multi-unit spiking activity across one shank of the MEA in three representative rats during natural sensory transmission. Warmer colours indicate more spikes per unit time; cooler colours indicate fewer spikes. Black arrows above the plots indicate times of innocuous light touch of the glabrous skin of the ipsilateral hindpaw; larger, red arrows indicate times of noxious pinch of the same receptive field. Periods between arrows depict spontaneous neural transmission (i.e. no induced sensory transmission or ISMS). Note the clear increase in the amount and anatomical spread of spiking activity during induced sensory transmission in the animals with SCI (and in particular SCI-NP) relative to the neurologically intact animal. This phenomenon is a hallmark of sensory hyperexcitability post-SCI. (**B**) Raster plot from a single rat depicting spiking activity of individual well-isolated neurons before, during and after VH ISMS. Red arrows above the subplots indicate times of noxious pinch of the glabrous skin of the ipsilateral hindpaw. Neurons shaded in blue were determined to be NS and neurons shaded pink were WDR. VH ISMS was delivered on the bottom-most electrode (highlighted via red box) during the times indicated above the raster plots. Black circles highlight the reduction of sustained discharge in response to instances of noxious peripheral stimuli that was often evident following ISMS.

In neurologically intact rats, brief periods of movement-targeted ISMS—i.e. ISMS delivered in the VH in vicinity of spinal motor pools—can immediately and persistently modulate intraspinal nociceptive transmission.^10^ However, it is difficult to generalize these findings to sensorimotor networks below an SCI given the maladaptive plasticity and profound alterations in neural excitability that occur during the chronification process. Such excitability changes are illustrated in Fig. 2A, which shows spinal responses to nociceptive and non-nociceptive sensory transmission in a representative rat without SCI (left), a rat with chronic SCI but no behavioural signs of SCI-NP (middle) and a rat with chronic SCI-NP (right). Increased discharge rates are clearly visible in the two animals with chronic SCI, as indicated by a shift towards warmer tones in the colour spectrum; further exacerbations are evident in the animal with SCI-NP. Additionally, the spatial distribution of spinal responses to sensory feedback increases dramatically in the animals with chronic SCI, consistent with an overall state of dorsal horn hyperexcitability due to a lack of descending neuromodulatory drive.

**Figure 3:**
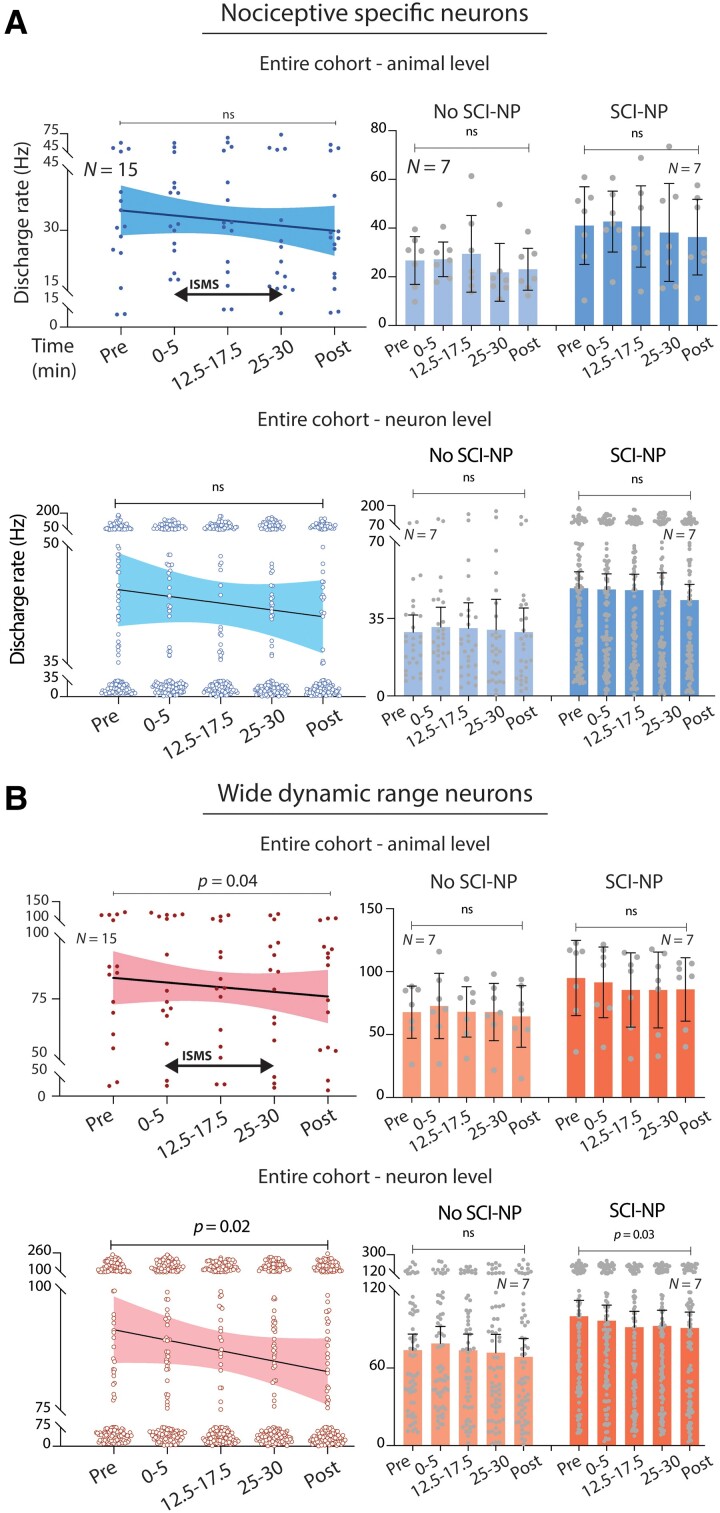
**Responsiveness of overall NS and WDR populations to VH ISMS.** (**A**, **B**, top) Animal-level pooled peak discharge rates for all NS and WDR neurons during noxious pinch of the ipsilateral hindpaw relative to ISMS duration (*N* = 15 rats). Bar plots are stratified by the presence or absence of SCI-NP. (**A**, **B**, bottom) Neuron-level peak discharge rates for NS (**E**) and WDR (**F**) neurons. Left plot in each panel pools all neurons from all animals; bar plots are stratified by whether the neurons were drawn from animals with or without SCI-NP. Linear regression lines of best fit are shown with accompanying 95% confidence bounds. One-way repeated measures ANOVAs with Bonferroni multiple comparisons correction were used to investigate potential differences in discharge rate across time point.

Thus, we first asked whether an overall cohort-level neuromodulatory effect was present across the populations of NS and WDR neurons (respectively). Figure 2B depicts a raster plot of one electrode shank before, during and following a single 30-min trial of movement-targeted ISMS in an animal with SCI-NP (the same animal as Fig. 2A, right). WDR neurons and NS neurons are indicated in red and blue, respectively. Pooling analogous data across all trials for a given animal and taking the animal-level grand mean, the discharge rate of NS neurons during periods of induced nociceptive transmission was statistically invariant to ISMS (*P* = 0.22; Fig. 3A, top left). In contrast, the animal-level grand mean for WDR neurons exhibited a statistically significant overall reduction in discharge rate during ISMS that remained depressed after cessation of stimulation (*P* = 0.04; Fig. 3B, top left). When animals were subgrouped based on the presence or­ absence of behavioural signs of SCI-NP, neither NS nor WDR discharge rate decreased significantly in response to nociceptive feedback as a function of ISMS duration (Fig. 3A, top right; Fig. 3B, top right). Ostensibly, the ‘loss’ of such an effect in WDR neurons was due to reduced statistical power in the subgroup analyses.

Although much of the biological variability in this experimental paradigm likely exists at the animal level, it was also of interest to understand if and how ISMS shapes neuron-level dynamics. Pooling all NS neurons from all animals, we found no effect of ISMS on discharge rate in response to nociceptive feedback (Fig. 3A, bottom left). The mean discharge rate of these neurons was likewise invariant to ISMS duration when stratified by the presence or absence of SCI-NP (Fig. 3A, bottom right). As with the animal-level grand means, however, increasing ISMS duration was associated with progressive depression of WDR responsiveness to nociceptive feedback (*P* = 0.02; Fig. 3B, bottom left). Interestingly, this trend was driven by neurons from animals with SCI-NP, which as a subgroup exhibited progressively depressed responses to nociceptive feedback as ISMS duration increased (*P* = 0.03; Fig. 3B, bottom right).

Previous work has shown that population-level trends (or lack thereof) often belie easily separable subgroups that reveal more granular insights into the state of spinal networks.^10,20,26-29^ Thus, we speculated that for both the NS and WDR populations, it would be possible to identify subpopulations of neurons that exhibited either potentiated or depressed discharge rates during nociceptive transmission. However, it was also reasonable to question whether the elevated state of dorsal horn excitability common to chronic SCI (e.g. Fig. 2A) would constrain the modulatory capacity of each population. Such an effect could lead to a lack of identifiable subpopulations, whether because discharge rates were indeed invariant to ISMS or because all neurons within a population converged towards a similar response to stimulation.

**Figure 4:**
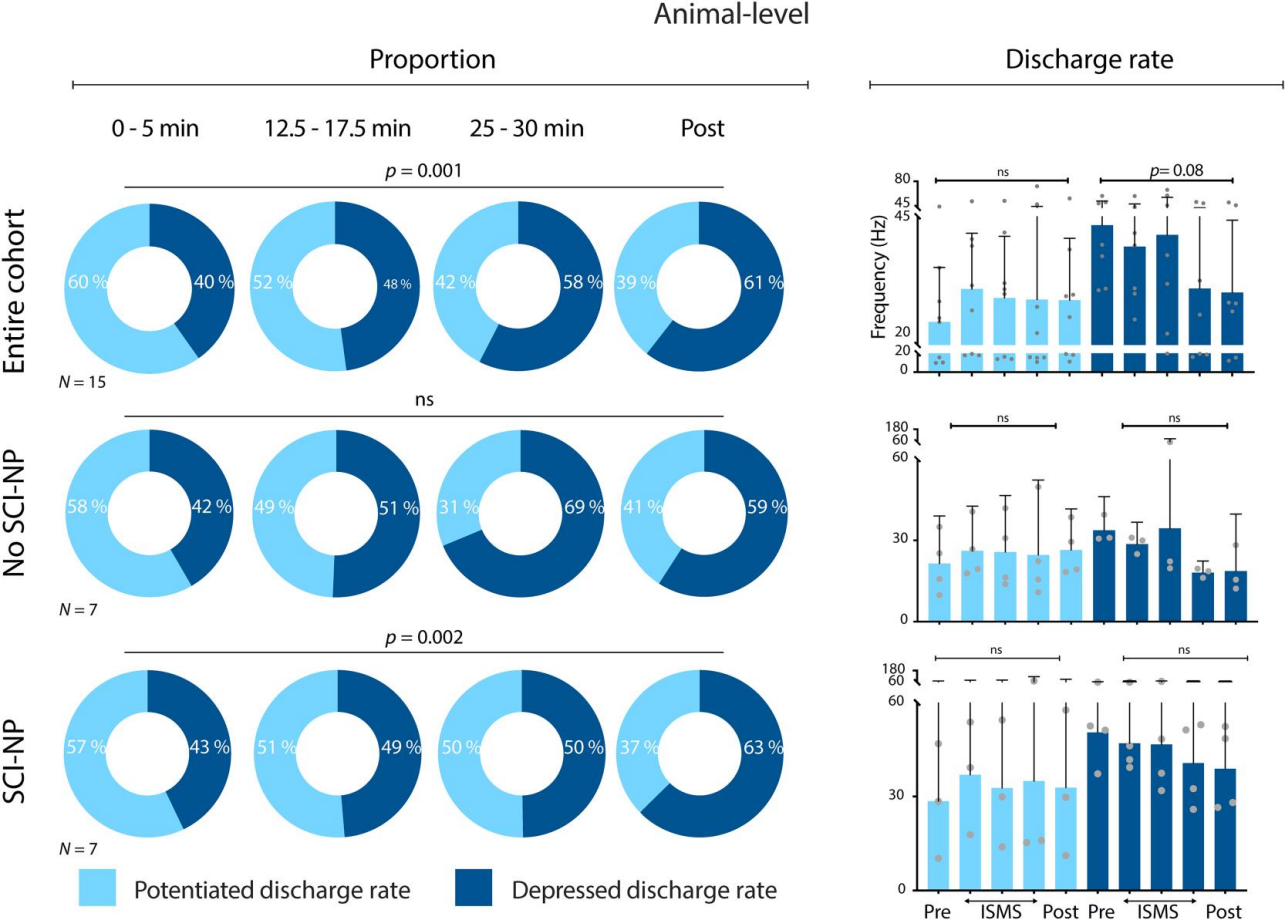
**VH ISMS drives progressive decreases in the animal-level responsiveness of NS neurons to noxious peripheral stimuli.** Animal-level proportion of identified NS neurons exhibiting potentiated (light) or depressed (dark) firing rates during induced nociceptive transmission relative to duration of ISMS. Top left panel: pooling across all rats (*N* = 15); middle left panel: subgroup of animals without SCI-NP (*N* = 7); bottom left panel: animals with SCI-NP (*N* = 7). Right panels depict the animal-level mean peak firing rate of NS neurons. All right panels are presented as mean peak discharge rate (+95% CI) of potentiated (light) and depressed (dark) NS neurons, respectively, during induced nociceptive transmission relative to ISMS. Potential changes in the proportion of depressed versus potentiated neurons were assessed via two-way repeated measures ANOVA with Bonferroni correction for multiple comparisons, while potential changes in discharge rate across time were assessed via one-way repeated measures ANOVA with Bonferroni correction for multiple comparisons.

**Figure 5:**
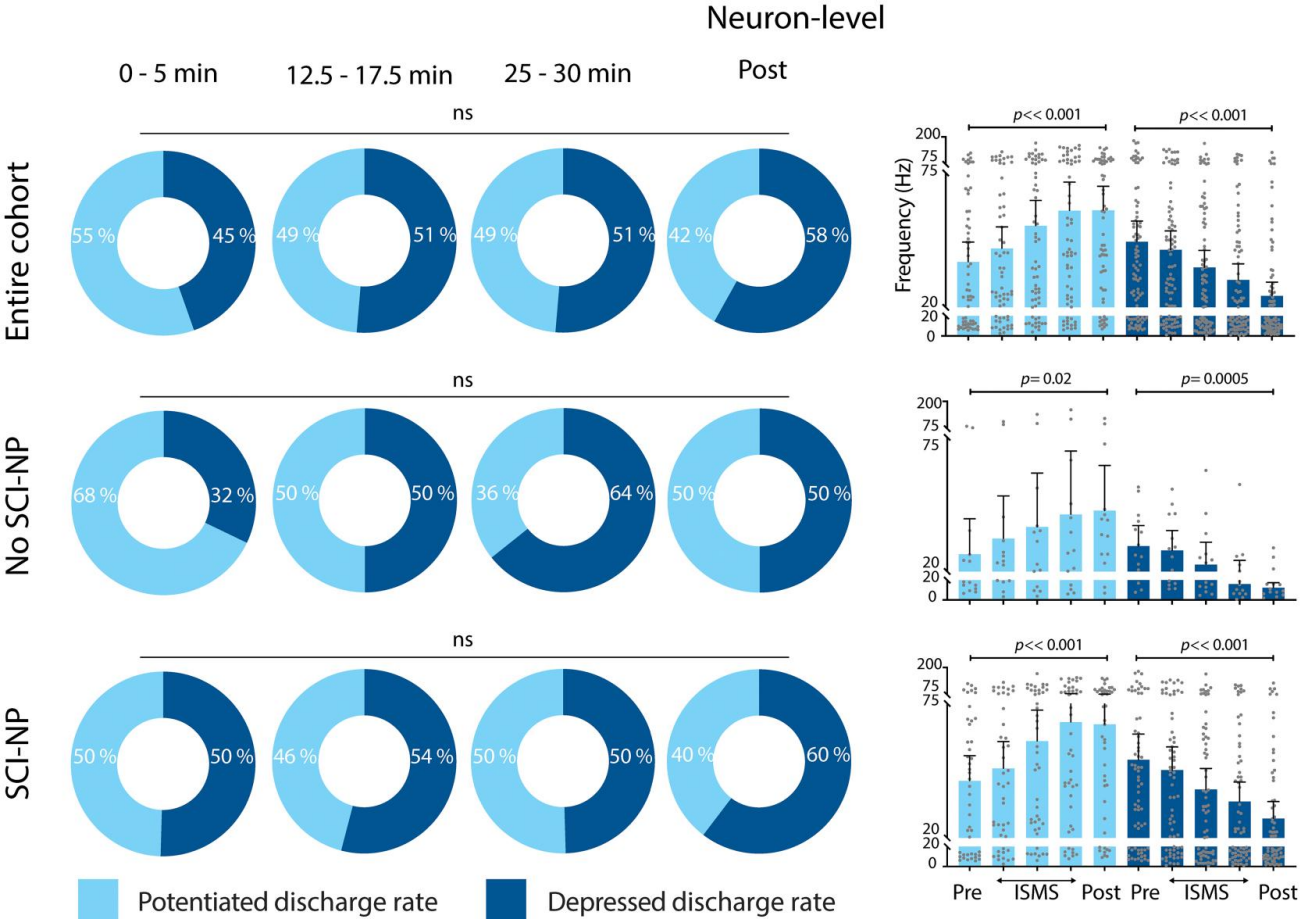
**VH ISMS modulates the firing rate, but not the overall proportion, of discrete NS neurons during noxious peripheral stimuli.** Neuron-level proportion of identified NS neurons exhibiting potentiated (light) or depressed (dark) firing rates during induced nociceptive transmission relative to duration of ISMS. Top left panel: pooling across all rats (*N* = 15); middle left panel (*N* = 7): subgroup of animals without SCI-NP; bottom left panel: animals with SCI-NP (*N* = 7). Right panels depict the neuron-level mean peak firing rate of NS neurons. All right panels are presented as mean peak discharge rate (+95% CI) of potentiated (light) and depressed (dark) NS neurons, respectively, during induced nociceptive transmission relative to ISMS. Potential changes in the proportion of depressed versus potentiated neurons were assessed via two-way repeated measures ANOVA with Bonferroni correction for multiple comparisons, while potential changes in discharge rate across time were assessed via one-way repeated measures ANOVA with Bonferroni correction for multiple comparisons.

**Figure 6:**
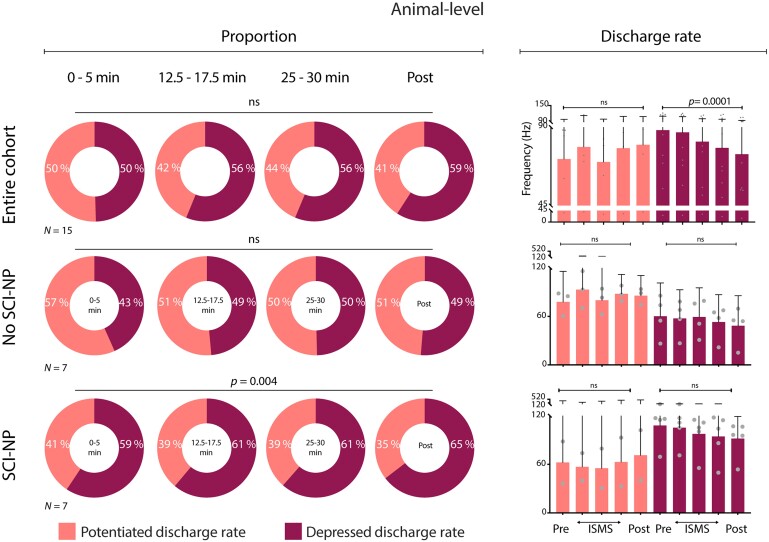
**VH ISMS preferentially decreases the animal-level responsiveness of WDR neurons to noxious peripheral stimuli.** Animal-level proportion of identified WDR neurons exhibiting potentiated (light) or depressed (dark) firing rates during induced nociceptive transmission relative to duration of ISMS. Top left panel: pooling across all rats (*N* = 15); middle left panel: subgroup of animals without SCI-NP (*N* = 7); bottom left panel: animals with SCI-NP (*N* = 7). Right panels depict the animal-level mean peak firing rate of WDR neurons. All right panels are presented as mean peak discharge rate (+95% CI) of potentiated (light) and depressed (dark) NS neurons, respectively, during induced nociceptive transmission relative to ISMS. Potential changes in the proportion of depressed versus potentiated neurons were assessed via two-way repeated measures ANOVA with Bonferroni correction for multiple comparisons, while potential changes in discharge rate across time were assessed via one-way repeated measures ANOVA with Bonferroni correction for multiple comparisons.

To address this question, we attempted to divide the NS and WDR neurons into subpopulations that were either potentiated by ISMS (post-ISMS discharge rate > pre-ISMS discharge rate) or depressed by ISMS (post-ISMS discharge rate <pre-ISMS discharge rate) (Figs 4 and 5 for NS neurons; Figs 5 and  6 for WDR neurons). Assignment of a neuron to the ‘potentiated’ or ‘depressed’ category was based solely on that neuron's post-ISMS mean discharge rate relative to its pre-ISMS mean discharge rate during nociceptive transmission; changes in discharge rate ‘during’ ISMS did not influence the classification. Thus, it was possible that a neuron coded as potentiated based on its pre/post discharge rates could exhibit ‘decreased’ discharge rates during ISMS. Likewise, a neuron coded as depressed could have exhibited increased discharge rates during ISMS, even though its post-ISMS rates were lower than its pre-ISMS rates.

This approach was chosen to avoid biasing the entire population-level ‘during-ISMS’ statistics. If neurons were reclassified at each time point during ISMS, some neurons would oscillate between the potentiated and depressed groups as ISMS continued. This would force a divergence between the two subpopulations that could inflate the statistical analyses. However, because of this choice, our estimates of the proportion of neurons with decreased discharge rates during ISMS and the mean discharge rate of the depressed subpopulation at each time point are likely an underestimate of the instantaneous dynamics of the population.

### NS neurons

Again pooling across all trials for a given animal and taking the animal-level grand mean, the proportion of NS neurons depressed by ISMS increased as a function of stimulation duration (*P* = 0.001; Fig. 4, top left). The average discharge rate of these neurons appeared qualitatively to decrease, but the significance of any such trend was equivocal (*P* = 0.08; Fig. 4, top right). Notably, however, the proportion of NS neurons exhibiting depressed firing rates during ISMS persisted effectively unchanged following discontinuation of ISMS. This outcome was unexpected, as the proportion of ISMS-depressed NS neurons in a recent report of neurologically intact rats exhibited a ∼25% decrease upon cessation of 30 min of stimulation. More surprisingly still, the proportion of NS neurons exhibiting persistent depression after discontinuation of ISMS was comparable in animals with chronic SCI (61%) to that reported in animals without neurological injury (55%).^10^ Together, these findings reveal unexpectedly robust carryover effects.

Regarding the subpopulation of NS neurons potentiated by ISMS, three features bear noting. First, the average peak discharge rate of these neurons was realized within the first 5 min of ISMS and remained unequivocally stable for the duration of stimulation (*P* = 0.27; Fig. 4, top right). This finding implies that ISMS did not contribute to unintended windup of nociceptive transmission through NS neurons, which could have exacerbated the behavioural consequences of SCI-NP. Second, the number of animals in which the majority of NS neurons were depressed by ISMS was more than double the number of animals in which the majority were potentiated (9 versus 4). Third, the average peak discharge rate of potentiated NS neurons was lower than that of the complementary subpopulation depressed by ISMS. Together, these findings reinforce the conclusion that ISMS did not drive an overall increase in net nociceptive transmission.

Next, we repeated these analyses when stratifying by the presence or absence of SCI-NP. We find that the significant cohort-level effect of ISMS duration on the proportion of depressed NS neurons was attributable in large part to animals with SCI-NP, who as a subgroup also exhibited a significant increase in the proportion of depressed NS neurons with ISMS duration (*P* = 0.002; Fig. 4, bottom left). By comparison, animals without SCI-NP did not exhibit a significant change in the proportion of depressed or potentiated NS neurons over this span (Fig. 4, middle left). Using neuron rather than animal as the grouping variable, there was no apparent effect of ISMS on the proportion of NS neurons potentiated or depressed, whether in SCI-NP or not (Fig. 5, left plots). However, there was a significant effect of ISMS duration on discharge rate for both subpopulations (Fig. 5, right plots).

### WDR neurons

Averaging across all animals, the response of WDR neurons to nociceptive sensory feedback was evenly divided between potentiated and depressed subpopulations during the first 5 min of ISMS (Fig. 6, top left). Unlike NS neurons, however, the highest proportion of ISMS-depressed WDR neurons (56%) was realized within the first 10–15 min of stimulation and remained constant thereafter. This finding suggests that the depressive effect of ISMS on WDR responses did not habituate over the course of stimulation, a conclusion supported by the observation that the animal-level peak discharge rate progressively decreased throughout the duration of ISMS (*P* = 0.0001; Fig. 6, top right). The proportion of WDR neurons depressed by ISMS also remained effectively unchanged following discontinuation of stimulation. Although potentially lower than has been previously reported in neurologically intact animals (60% versus 73%),^10^ the number of animals exhibiting net depression of WDR responses to nociceptive transmission was more than 2-fold higher than animals exhibiting a net potentiation (10 versus 4).

We then characterized WDR responsiveness to nociceptive feedback when stratifying by the presence or absence of SCI-NP. These analyses revealed that animals without SCI-NP did not exhibit a significant overall difference in the number of potentiated or depressed neurons with respect to ISMS or a progressive shift towards a greater proportion of one relative to the other during ISMS (Fig. 6, middle left). Likewise, animal-level discharge rate in this group was not significantly associated with ISMS duration (Fig. 6, middle right). In contrast, animals with SCI-NP exhibited significantly more neurons with depressed responses to nociceptive feedback than neurons with potentiated responses (*P* = 0.004; Fig. 6, bottom left). This effect was stable over time, neither shifting in proportion nor discharge rate as a function of ISMS duration (Fig. 6, bottom left and right).

**Figure 7:**
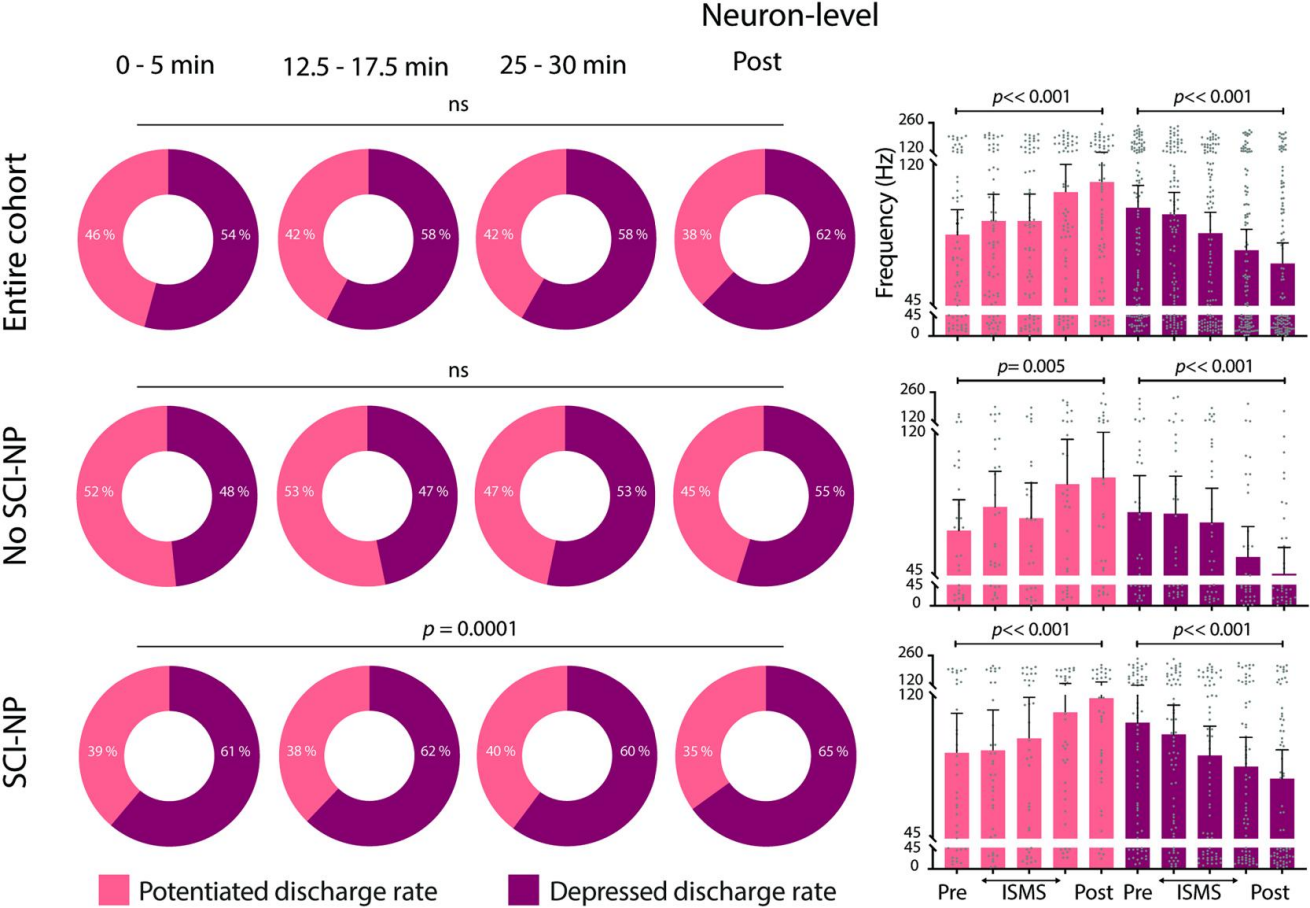
**VH ISMS preferentially decreases the neuron-level responsiveness of WDR neurons to noxious peripheral stimuli in animals with SCI-NP.** Neuron-level proportion of identified NS neurons exhibiting potentiated (light) or depressed (dark) firing rates during induced nociceptive transmission relative to duration of ISMS. Top left panel: pooling across all rats (*N* = 15); middle left panel: subgroup of animals without SCI-NP (*N* = 7); bottom left panel: animals with SCI-NP (*N* = 7). Right panels depict the neuron-level mean peak firing rate of NS neurons. All right panels are presented as mean peak discharge rate (+95% CI) of potentiated (light) and depressed (dark) NS neurons, respectively, during induced nociceptive transmission relative to ISMS. Potential changes in the proportion of depressed versus potentiated neurons were assessed via two-way repeated measures ANOVA with Bonferroni correction for multiple comparisons, while potential changes in discharge rate across time were assessed via one-way repeated measures ANOVA with Bonferroni correction for multiple comparisons.

Neuron-level trends in the proportion of WDR cells exhibiting potentiated versus depressed responses to ISMS mirrored the animal-level findings. Specifically, when considering all WDR neurons from the cohort as a whole and when considering only those drawn from animals without SCI-NP, we found no statistical dependence on ISMS (Fig. 7, top and middle left panels). In animals with SCI-NP, significantly more WDR neurons exhibited depressed responses to nociceptive feedback than exhibited potentiated responses (*P* = 0.004; Fig. 7, bottom left). Interestingly, this difference was realized within the first 5 min of ISMS and was maintained unchanged throughout the duration of stimulation, distinguishing the impact of ISMS on WDR neurons from that of NS neurons. Neuron-level trends in discharge rate as a function of ISMS duration were significant both for increased rates and decreased rates, respectively, and regardless of whether the all neurons were pooled form the entire cohort or if they were subgrouped by the presence or absence of SCI-NP (Fig. 7, right).

Thus, considering also the net depressive effects of ISMS on the proportion of NS neurons—particularly in animals with SCI-NP—these findings establish a heretofore unreported multi-modal neuromodulatory effect of movement-targeted spinal stimulation: namely, concurrent depression of spinal nociceptive transmission preferentially impacting animals with SCI-NP.

### Movement-targeted ISMS does not robustly modify spinal responsiveness to non-nociceptive sensory feedback

Previous studies have hypothesized that recruitment of non-nociceptive sensory afferent pathways is necessary (albeit insufficient) for electrical spinal stimulation paradigms to enhance voluntary movement and, separately, to reduce nociceptive transmission. In the context of motor control, recruitment of non-nociceptive sensory afferent pathways is presumed to augment or replace weakened or absent excitatory synaptic drive to motoneuron pools below the SCI.^2,5,7,30^ In the context of pain management, activation of low-threshold afferent pathways has been theorized to engage a gating-like mechanism that meters the transmission of nociceptive information through WDR neurons.^4,8^ Yet, it has also been reported in the uninjured spinal cord that sub-motor threshold ISMS delivered in the VH exerts a negligible impact on WDR responsiveness to non-nociceptive cutaneous and/or proprioceptive sensory feedback.^10^

**Figure 8:**
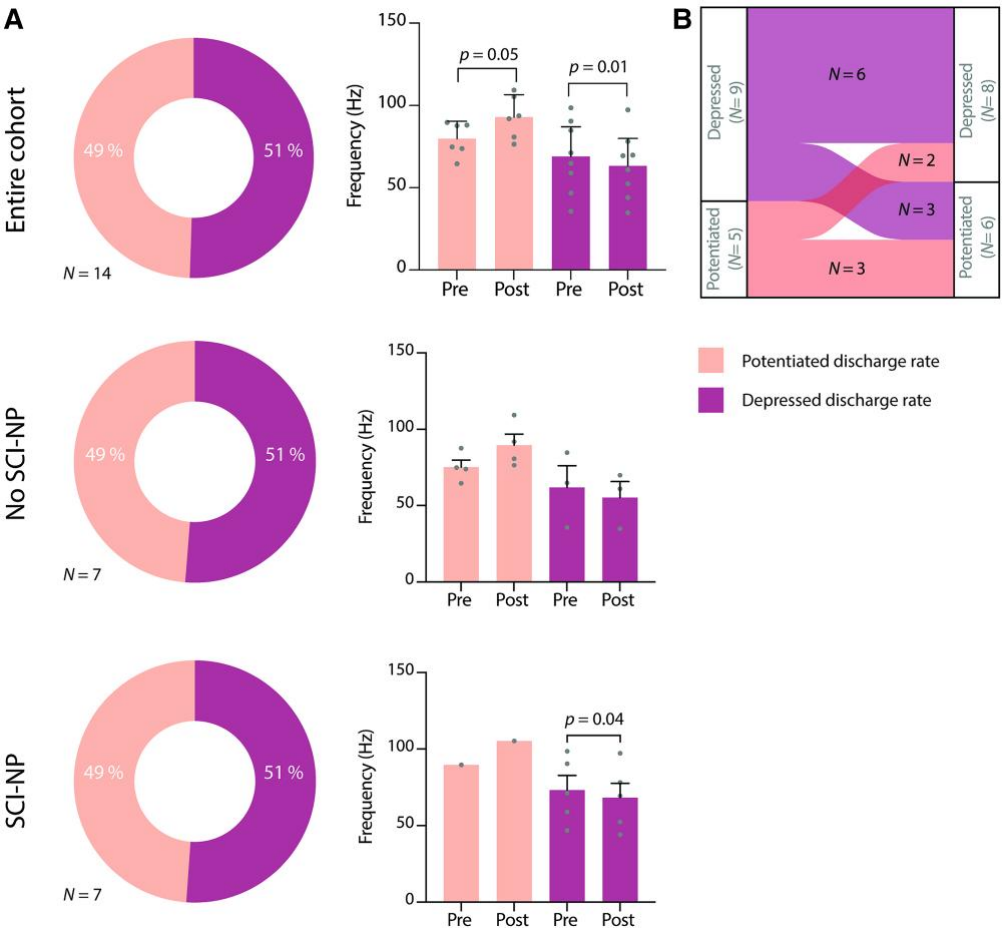
**VH ISMS exhibits an unexpectedly diminished capacity to modulate non-nociceptive cutaneous transmission relative to nociceptive transmission.** (**A**) Percentage of WDR neurons (average of 11 neurons per rat; *N* = 14 rats) with potentiated (lighter) or depressed (darker) discharge rates during innocuous touches of the ipsilateral hindpaw after 30 min of ISMS relative to before ISMS. Top left: pooling across the entire cohort of animals (*N* = 14); middle left: subgroup of animals without SCI-NP (*N* = 7); bottom left: subgroup of animals with SCI-NP (*N* = 7). Right panels: mean peak WDR discharge rate (+95% CI) in response to innocuous touches of the ipsilateral hindpaw immediately prior to and following 30 min of ISMS, respectively. *P*-values derived from paired, two-tailed *t*-tests. (**B**) Alluvial plot depicting cases in which the modulatory effects of ISMS shifted from potentiation to depression (or vice versa) when transitioning between sensory modes.

It was unclear whether movement-targeted ISMS alters the responsiveness of WDR neurons to non-nociceptive cutaneous feedback below a chronic SCI. Therefore, we characterized the discharge rate of WDR neurons in response to such feedback before and after 30 min of ISMS (in 159 discrete WDR neurons across 14 animals). Across the entire population of identified WDR neurons, we found no difference in discharge rate following ISMS compared with the corresponding pre-ISMS baseline (pre-ISMS: 73.55 ± 4.83 Hz; post-ISMS: 76.04 ± 6.02 Hz). Consistent with this observation, the proportion of WDR neurons exhibiting potentiated versus depressed responses to non-nociceptive sensory feedback following ISMS were indistinguishable (potentiated: 49%; depressed: 51%; Fig. 8A, top left).

These findings suggested that movement-targeted ISMS exerted modality-specific modulatory actions that preferentially influenced nociceptive transmission relative to non-nociceptive transmission (as opposed to non-specifically modifying overall WDR excitability). To determine if the modulatory actions of ISMS on multi-modal sensory transmission were more nuanced than they initially appeared, we again attempted to subdivide the neuron-level population into animal-level groups in which the majority of WDR neurons identified in each animal was either potentiated or depressed by ISMS. This manipulation revealed statistically significant, albeit modest (∼12%), increases and decreases (respectively) in discharge rate associated with ISMS (*P* = 0.05 for potentiated; *P* = 0.01 for depressed; Fig. 8A, top middle). When further stratified by the presence or absence of SCI-NP, the proportion of potentiated versus depressed neurons was unchanged: 50/50 for both groups (Fig. 8A, middle, bottom), although ISMS in animals with SCI-NP was associated small decrease in discharge rate during innocuous sensory feedback (*P* = 0.04; Fig. 8A, bottom middle).

We then sought a different approach to understand the apparent modal specificity of ISMS. We reasoned that modal specificity could manifest in either of two ways: (i) differential modulatory actions across modes of sensory transmission (e.g. one mode potentiated and one mode depressed) or (ii) an enhanced modulatory capacity during one mode of sensory transmission relative to another. To this end, we first determined the number of animals in which the net modulatory action of ISMS shifted from potentiation to depression (or vice versa) when transitioning between nociceptive and non-nociceptive transmission. In total, five (of 14) animals exhibited such behaviour, with three animals having depressed responses to nociceptive transmission and potentiated responses to non-nociceptive transmission, and 2 animals manifesting the opposite trend (Fig. 8B).

Next, we quantified the average magnitude of discharge rate changes associated with ISMS during nociceptive and (separately) non-nociceptive transmission in each animal. Across all animals, and regardless of whether a given modality was depressed or potentiated, we found a 3-fold greater change in discharge rate during nociceptive transmission compared with non-nociceptive transmission (not shown). In only one animal was the magnitude of change in non-nociceptive transmission demonstrably greater than during nociceptive transmission, and in two animals, the change in ISMS-associated discharge rate was indistinguishable across modes (both cases in which non-nociceptive transmission was potentiated on balance).

These findings could not be explained by a ‘floor’ effect, wherein discharge rates during non-nociceptive transmission prior to ISMS were low enough that they could not be reduced further. Indeed, the mean discharge rates across animals for WDR responses to nociceptive and non-nociceptive transmission were indistinguishable prior to ISMS (*P* = 0.24; two-tailed *t*-test). Thus, in 11 of 14 animals, the modulatory actions of ISMS were less robust during non-nociceptive transmission than during nociceptive transmission, leading to the conclusion that the apparent modal specificity of ISMS is predominantly the result of preferential modulation of nociceptive transmission and not differential modulation of each mode.

### Overall dorsal horn excitability is not changed by movement-targeted ISMS despite its anti-nociceptive actions

Finally, it was reasonable to envision a scenario in which broad shifts in overall or regional spinal excitability associated with ISMS led to differential modulation of nociceptive versus non-nociceptive responsiveness. Such a finding would argue against the explanation that ISMS intrinsically (albeit enigmatically) drives modality-specific effects and would instead suggest that this observation was an epiphenomenon related to a complex, emergent pattern of network activity.

**Figure 9:**
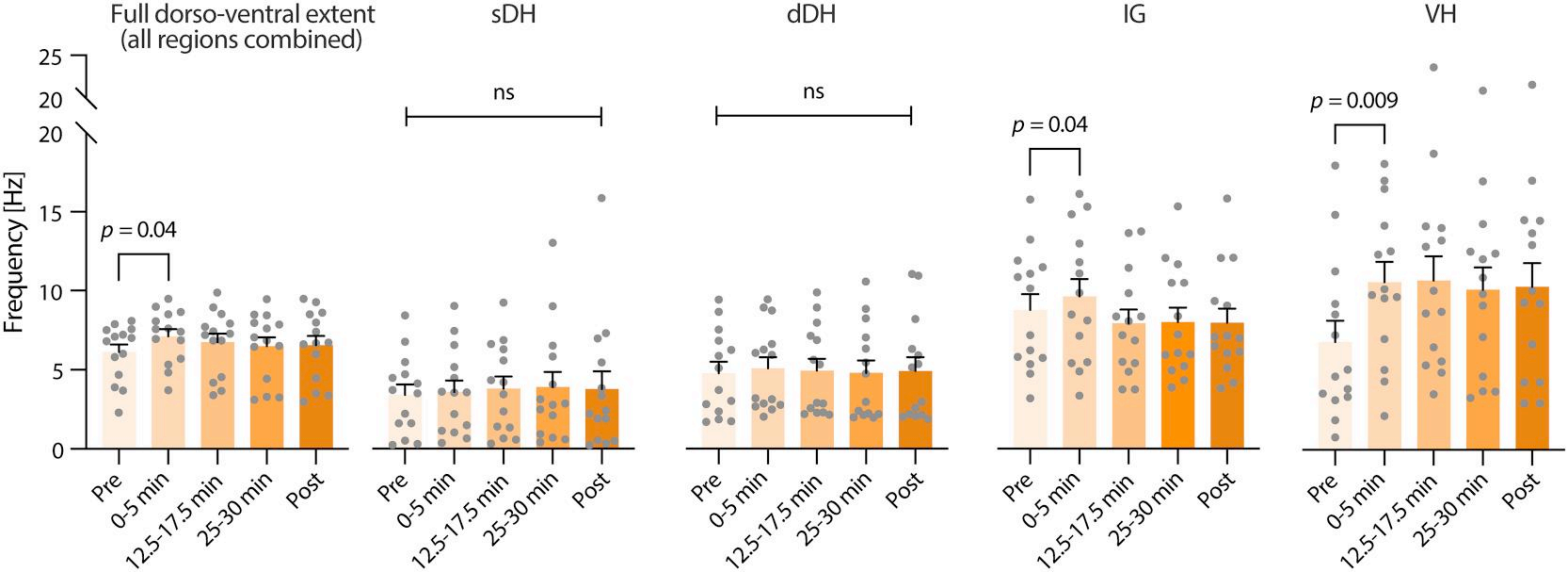
**Modulatory actions of ISMS on nociceptive transmission are not accompanied by broad shifts in segment-wide neural excitability.** Bar plots depict the mean discharge rate of all active neurons (NS, WDR and NC) per animal before, during and after VH ISMS. ISMS was delivered within the VH for 30 min at sub-motor threshold intensity. No changes in discharge rate are evident in the sensory-dominant sDH or dDH, and the IG, a sensorimotor integrative region, experiences only a transient increase in excitability upon stimulation onset. By comparison, VH discharge rate is increased more robustly as expected for ventral ISMS. Error bars depict mean + sem; *N* = 14 animals; one-way repeated measures ANOVA with Bonferroni correction.

To determine if diffuse, pseudorandom changes in neural excitability were associated with ISMS, we considered the population discharge rate of ‘all’ identified neurons from each animal—NS, WDR and NC—during periods of spontaneous neural transmission before and after ISMS. We began by investigating the composite discharge rate spanning the dorsoventral extent of the L5 spinal segment (i.e. including the sDH, dDH, IG and VH) to gauge neural excitability at the broadest level. After a modest but statistically significant segment-wide increase in discharge rate during the first 5 min of ISMS (pre-ISMS: 6.11 ± 0.48 Hz; first 5 min of ISMS: 7.12 ± 0.45; *P* = 0.003; Fig. 9), the net increase in excitability habituated and became indistinguishable from pre-ISMS baseline levels for the remainder of stimulation and following cessation of ISMS.

Next, we explored the potential impact of ISMS on region-specific excitability. We found no changes in mean spontaneous population discharge rate before or after ISMS in either the sDH or the dDH (Fig. 9), regions predominantly associated with transmission of nociceptive and non-nociceptive cutaneous sensory feedback. In contrast, significant differences in population discharge rate were evident within the first 5 min of ISMS in the IG (pre-ISMS: 8.76 ± 1.00 Hz; first 5 min of ISMS: 9.63 ± 1.12 Hz, *P* = 0.04) and the VH (pre-ISMS: 6.82 ± 1.35 Hz; first 5 min of ISMS: 10.60 ± 1.30 Hz, *P* = 0.009) (Fig. 6). In the IG, population discharge rate rapidly returned to its pre-ISMS baseline. In the VH, by comparison, mean population discharge rate remained elevated for the duration of ISMS and afterwards, but did not achieve statistical significance due to an accompanying increase in dispersion of the discharge rates across animals. The increased discharge rate in the VH is expected given that ISMS was delivered in this region and would be consistent with enhanced voluntary movement as reported previously using these parameters.^21^ Ultimately, the results of these analyses indicated that it is highly unlikely that a net shift in spinal excitability contributed to the preferential depression of nociceptive rather than innocuous sensory transmission.

## Discussion

We demonstrated in a rat model of SCI that the responsiveness of spinal neurons integral to the development and persistence of SCI-NP can be reduced by ISMS delivered directly to the VH of the same spinal segment. We also show that these depressive actions are relatively specific to nociceptive transmission, as spinal responsiveness to non-nociceptive transmission was largely unchanged by stimulation while motor-related transmission was potentiated.

It was not clear *a priori* whether sub-motor threshold ISMS parameterized to enhance voluntary movement would be capable of altering neural transmission in sensory networks already made pathologically overactive by SCI (e.g. as in Fig. 2A and Bandres *et al*.^29^). In fact, the extent to which the implicated networks can even be modulated in a therapeutically relevant way, regardless of the specific approach used to affect such a change, remains an active area of research in the field. After all, maladaptive neural activity was allowed to develop unchecked and to chronify naturally, resulting in a ‘worst case scenario’ for plasticity-promoting interventions. Whereas pre-clinical and clinical studies have repeatedly demonstrated the ability of motor-dominant circuits below a lesion to reorganize,^21,31-37^ neural transmission in spinal pain pathways has been comparatively difficult to shape. Indeed, interventions explicitly designed to promote functionally relevant neural plasticity as well as those that attempt to pharmacologically rebalance spinal excitability have been met with variable success—especially when initiated after establishment of the pain state.^38,39^

Thus, it was reasonable to speculate that reinforcement of inappropriate patterns of neural transmission during the chronification process, combined with a lack of descending inhibition, would have rendered the spinal environment impermissive for allowing focal, low-amplitude stimulation delivered distantly in the VH to modulate transmission in spinal pain pathways. Alternatively, it was also reasonable to predict that injection of *any* current into the spinal cord below the lesion would increase segment-wide excitability, exacerbating overactive spinal responses to sensory feedback by trans-synaptically depolarizing many of the same neurons (or their inputs) responsible for *de novo* development of the hyperexcitable state. Yet, despite this potent backdrop of excitability, ISMS rapidly modulated transmission in spinal pain pathways, resulting in depressed NS and WDR responses to nociceptive transmission. Although the present stimulation parameters were not designed to promote enduring plasticity nor was the study designed to systematically characterize the time course of carryover effects, that we observed persistent excitability changes at all—and with only a single, relatively short session of ISMS—indicates both that the capacity for plasticity remains and that it has the potential to be unlocked via spinal stimulation.

Scientifically, a particularly intriguing aspect of these findings was the differential impact of ISMS on spinal networks predominantly sub-serving different functions. Potentiated transmission in motor-dominant VH networks was evident as expected (e.g. Fig. 6), but this excitation was coupled with net depression of nociceptive transmission and effectively unchanged transmission of non-nociceptive cutaneous sensory feedback. More interestingly, these actions were defined across functional but not anatomical boundaries. Indeed, the overall invariance of spinal segment-level excitability during ISMS could not be explained by opposing excitatory and inhibitory actions across anatomical regions, effectively cancelling the appearance of a segment-wide effect. For example, depressed nociceptive transmission was evident in NS and WDR neurons identified in the IG and VH, regions that experienced a net increase in excitability during ISMS, whereas the responsiveness of NS and WDR neurons in the sDH and dDH was also depressed by ISMS, yet these anatomical regions saw no net change in excitability.

It is possible that the balance of excitation and inhibition would shift under awake, behaving conditions. For example, it has been demonstrated that appropriate recruitment of pre-motor proprioceptive networks is essential for providing the inhibition necessary to sculpt the diffuse excitation mediated by epidural spinal stimulation.^2,5^ Although it is incompletely understood whether analogous engagement of supporting circuits could play a role in shaping the balance of excitation and inhibition within sensory-dominant networks, it would seem a plausible, if not likely, scenario; many examples of natural sensory gating phenomena have been reported.^40-43^ In fact, it is possible that such a mechanism could have contributed to what phenomenologically appeared to be heterosynaptic plasticity in WDR neurons, wherein long-term depression of high-threshold inputs was contrasted by negligible changes at low-threshold synapses converging onto the same cells. Regardless, if borne out by future work in awake, behaving animals, the modulatory actions observed here would distinguish sub-motor threshold ISMS from epidural spinal stimulation and provide a powerful new platform for developing multi-modal rehabilitation therapies.

Also intriguing was the observation that the depressive effects of ISMS appear to be more robust in animals with SCI-NP than those without. The mechanisms underlying this differential effect are not intuitively obvious. Indeed, spinal contusion parameters were identical for all animals and the resulting motor impairments were qualitatively similar (with the exception of one animal that developed near complete bilateral paralysis). It is possible that slight variations in anatomy or the location of the impactor tip resulted in different mixtures of remaining viable pathways between animals with SCI-NP and those without. If these were predominantly endogenous neuromodulatory pathways that would typically exert a net depressive effect on nociceptive transmission (e.g. noradrenergic coeruleospinal pathways), ISMS may have serendipitously capitalized on this distinction given its ability to recruit axons and fibres of passage at lower current intensities than cell bodies. Such a systematic effect across subgroups seems unlikely to have resulted from the contusion itself, however. Nevertheless, future work is warranted to determine whether the integrity of neuromodulatory pathways was a component of the observed effects. If so, it could point to adjuvant therapies that could increase the efficacy of ISMS to enhance motor output and to reduce pain-related transmission.

Two particular aspects of the ISMS protocol also bear noting. First, stimulation was delivered in an open-loop fashion rather than in an activity-dependent manner. That is, delivery of ISMS was uncorrelated with ongoing neural activity. As a result, the ISMS paradigm made no explicit attempts to restore or reinforce natural, appropriate patterns of neural transmission in the modulated networks. Previously, it has been shown that activity-dependent spinal stimulation leads to functional gains that far exceed those afforded by open-loop stimulation, presumably by more optimally engaging circuits below the injury to promote beneficial neural plasticity.^21,32,44,45^

Pre-clinical models also indicate that the enhanced efficacy of activity-dependent stimulation is not immediate. Rather, the effects of activity-dependent stimulation paradigms diverge from open-loop paradigms several weeks into an intervention, continuing to enhance function beyond open-loop approaches over time and leading to therapeutic gains that can persist for weeks after discontinuation of stimulation.^21,45^ Together, these observations suggest that the plasticity observed here may be a conservative representation of what could be achieved with activity-dependent stimulation and/or in the context of a directed rehabilitation intervention.

Second, modulation of sensory neurons was realized with ISMS ‘intended to enhance movement’. Indeed, the approach was not configured to directly alter the input–output function of dorsal horn neurons, for example by juxtaposing the stimulating electrode in a region dense with identified NS or WDR neurons. Instead, ISMS was delivered in the VH adjacent to spinal motor pools. This distinction suggests that it may be possible to purposefully engineer a spinal stimulation-based intervention to deliver multi-modal therapeutic benefits. Here, those benefits centred upon simultaneously rebalancing the pathologic sensory and motor transmission that contribute to debilitating motor impairments and SCI-NP. However, the high interconnectivity and diversity of physiological functions mediated by spinal networks suggest that other combinations could be envisioned. Paradigms intended to simultaneously enhance motor output while restoring bowel, bladder or sexual function would address many of the issues routinely deemed most important by people living with SCI.^46-49^

Given that ISMS will require additional time to progress through the translational pipeline before potentially emerging as a viable clinical option, the logical question becomes how to accelerate introduction of these ideas into advanced yet currently available therapies. Fortunately, many of the results obtained with ISMS support the idea that epidural spinal stimulation may also be capable of delivering multi-modal benefits. Nominally, it is worth reiterating that clinically available epidural spinal stimulators were originally developed to manage medically refractory pain conditions, and the parameter sets and electrode montages used in movement rehabilitation applications closely mirror those employed by the pain field. In fact, it has traditionally been posited that the analgesic effects of paraesthesia-based spinal stimulation paradigms are mediated by mechanisms highly overlapping with those intended to enhance spinal motor output (although this interpretation is now understood to be incomplete).^2,4,5,7,8,10,30^ Thus, the literal ability of epidural spinal stimulation to modulate both sensory and motor networks is not in question.

A striking feature of these results was the specificity with which movement-targeted ISMS modulated spinal responsiveness to nociceptive transmission despite the broad spatial extent over which the identified neurons were located—particularly given that stimuli were delivered at sub-threshold intensity for evoking muscle contractions. This diffusivity implies that focally delivered stimulation may not be essential to achieve qualitatively similar sensory and movement-related effects to those observed here. At present, however, the majority of epidural stimulation paradigms to enhance movement use electrical current intensities supra-threshold for evoking contractions,^44,50,51^ whereas epidural stimulation for pain management remains sub-threshold for motor unit recruitment.^52,53^ Unlike supra-threshold stimulation, we did not observe net increases in non-nociceptive transmission via large-diameter afferents. Thus, to the extent that stimulation location(s) and target(s) will have implications for stimulation intensity and presumably for the ensuing modulatory actions, it will be critical to reconcile how to parameterize epidural spinal stimulation for multi-modal applications. One starting point could be renewed investigation of sub-motor threshold epidural stimulation,^9,54^ which is designed to augment volitional attempts to move rather than to produce the intended movement(s) directly.

In the immediate term, two additional considerations would accelerate translation of these results. First, ongoing or planned studies of epidural spinal stimulation intended to enhance voluntary movement could specifically incorporate assessments of pain perception and sensory acuity into their battery of outcome measures; there are many validated instruments for these purposes. While fewer such studies are ongoing, there is likewise much to be gained from incorporation of movement assessments into studies of epidural spinal stimulation for SCI-NP. Second, enrolment of people living with sensorimotor incomplete SCI, especially those living with SCI-NP, into trials of epidural spinal stimulation for rehabilitation of movement impairments would provide a wealth of insights. Purposeful, systematic acquisition of data pertaining to the off-target effects of epidural spinal stimulation will move the neuromodulation field towards conceptualization and development of therapies focusing on the complex, interrelated rehabilitation goals of people living with SCI.

## Data Availability

Data will be made freely available upon reasonable request to the corresponding author.
